# Metabolic Difference of CZ48 in Human and Mouse Liver Microsomes

**DOI:** 10.3390/ijms13055498

**Published:** 2012-05-08

**Authors:** Xing Liu, Albert DeJesus, Zhisong Cao, Dana Vardeman, Beppino Giovanella

**Affiliations:** CHRISTUS Stehlin Foundation for Cancer Research, 10301 Stella Link, Houston, TX 77025, USA; E-Mails: lliu@stehlin.org (X.L.); adejesus@stehlin.org (A.D.); dvardeman@stehlin.org (D.V.); bcg@stehlin.org (B.G.)

**Keywords:** metabolism, biotransformation, cytochromeP450, UDP-glucuronosyltransferases, microsomes, CZ48, camptothecin

## Abstract

CZ48, chemically camptothecin-20-*O*-propionate hydrate, is currently under clinical investigation. The kinetics of the metabolite camptothecin (CPT) formation and of CZ48 depletion in mouse and human liver microsomes in the presence or absence of NADPH was examined. The formation rate of camptothecin in human liver microsomes was significantly higher than that in mouse with mean *K*_m_s of 1.9 and 0.5 nM and *V*_max_s of 9.3 and 2.2 pmol/min/mg, respectively. However, the apparent intrinsic clearance (*V*_max_/*K*_m_) ratios for camptothecin in human and mouse liver microsomes were not significantly different from each other (4.9 *versus* 4.4) in the presence of NADPH. The depletion of CZ48 in human microsomes was four times faster with 4.55% of CZ48 remaining intact while in mouse 19.11% of the drug remained unchanged after 60 min. These results suggest that there is a remarkable species difference of CZ48 biotransformation between human and mouse. The depletion rate of CZ48 in human liver microsomes is considerably higher than that in the mouse.

## 1. Introduction

Metabolism studies are essential in the drug discovery and development process and are important to safety studies for the clinical development of drug candidates. Much progress has been made in recent years in understanding mechanisms of toxicities caused by drug metabolites and determining the numerous factors influencing individual exposure to products of drug biotransformation. The cytochrome P450 (CYP) superfamily is a large group of enzymes. CYPs are found to be the major enzymes involved in drug metabolism and biotransformation, accounting for approximately 75% of the total number of metabolic transformations [1]. Glucuronidation is increasingly recognized as an important clearance pathway in addition to that of P450 enzymes. The total contribution of P450 and UDP-glucuronosyltransferases in drug metabolism is reported to be more than 80% [2,3]. Hydrated crystalline camptothecin-20-propionate, CZ48, is under investigation for the treatment of cancer and is currently in phase 1 human clinical trials. This agent has shown remarkable anticancer activity and a lack of toxicity in nude mice. Differing from other camptothecin derivatives, CZ48 is stable in both mouse and human plasmas and the majority of the molecule remains in the intact lactone form. CZ48 has a huge favorable therapeutic index ranging from 2 to 40 when treating human tumors grown as xenografts in nude mice, while other camptothecin derivatives and conventional anticancer agents have only a very narrow therapeutic window of 1 to 1.2. We previously reported our results in Cancer Research in 2009 [4]. The phase 1 trial with this agent started in late 2008 and more than 30 patients have been treated since then and the trial continues. To date the MTD (Maximum Tolerated Dose) has not yet been reached and clinical trials are ongoing with cohorts of three. We have pharmacokinetically analyzed the blood samples of 27 patients and observed that the concentration of CZ48 is 10 or more times lower in human blood than in the mouse, while the concentration of camptothecin (CPT) is multiple folds higher in human blood than in the mouse (our unpublished results). The cause for this is probably that CZ48 has been biotransformed differently in the human body compared to the mouse. CPT is found to be the only identified metabolite of CZ48.

A number of papers in literature report that human liver microsomes catalyze the metabolisms of different types of drugs. Both human liver slices and microsomes can be used to study drug metabolism. Human liver microsomes are proven to be several times faster than slices [5–7], and clearance predictions are closer to *in vivo* values than are predictions from slices [8]. In this study, we examined the kinetics of the formation of the metabolite CPT and of the depletion of CZ48 in mouse and human liver microsomes for assessing the effect of species on the biotransformation of CZ48. The information gained concerning the species differences from this study will be greatly helpful for extrapolating results from animals to humans.

## 2. Results and Discussion

**Figure 1:**
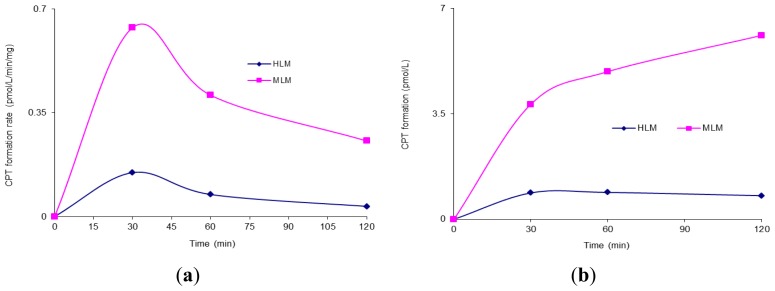
(**a**) Camptothecin (CPT) formation rate in human and mouse liver microsomes; (**b**) Accumulated formation of CPT in human and mouse liver microsomes. (**a**) (**b**)

The conversion of CZ48 to CPT within 2 h in human *versus* mouse liver microsomes was investigated by incubating the agent in HLM (human liver microsomes) and MLM (mouse liver microsomes), respectively, in the absence of NADPH. The results are shown in Figure 1.

Figure 1a shows the formation rate of the metabolite CPT in HLM and MLM, respectively, during the period of the incubation. Figure 1b summarizes the accumulated formation of CPT in these microsomes within 120 min. As shown in Figure 1b, the accumulative amounts of CPT formed in MLM and HLM were 6.11 ± 0.107 and 0.78 ± 0.13 pmol/L, respectively.

**Figure 2:**
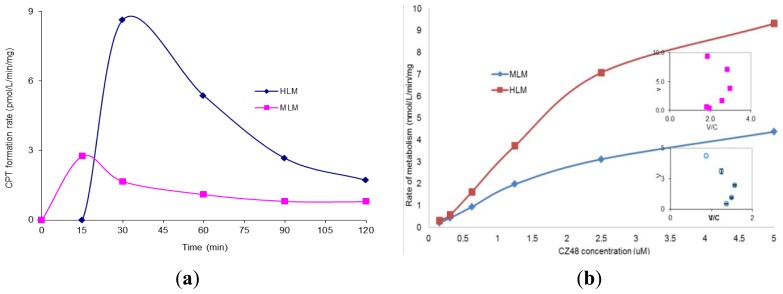
(**a**) CPT formation rate in human and mouse liver microsomes in the presence of NADPH; (**b**) The kinetics of the CPT formation in human and mouse liver microsomes. (**a**) (**b**)

The incubation of CZ48 in HLM and MLM in the presence of NADPH for 2 h was subsequently performed and the CPT formation rate results are shown in Figure 2a. The relationship between the metabolism rate and the concentrations of CZ48 in HLM within the range of 0.15 to 5 μM was studied and plotted as shown in Figure 2b with *K*_m_ 5.24 ± 0.23 μM and *V*_max_ 19.65 ± 0.026 nmol/min/mg for HLM and *K*_m_ 0.46 ± 0.13 μM and *V*_max_ 2.20 ± 0.06 nmol/min/mg for MLM.

**Figure 3:**
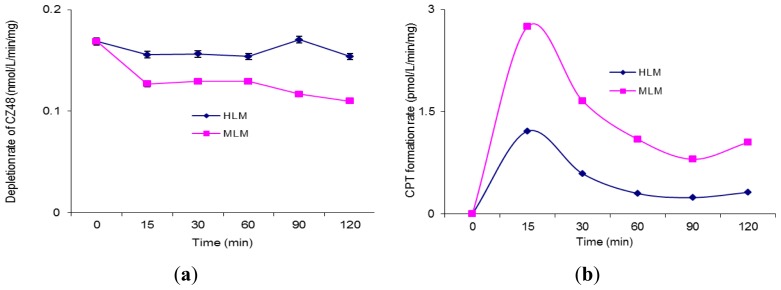
(**a**) Depletion rate of crystalline camptothecin-20-propionate (CZ48) in human and mouse liver microsomes in the presence of UDPGA. (**b**) CPT formation rate in human and mouse liver microsomes in the presence of UDPGA. (**a**) (**b**)

The incubation of CZ48 in HLM and MLM in the presence of UDPGA was also performed to study the effect of UDP-glucuronosyltransferases (UGTs) on the metabolism of CZ48 in humans and the mouse. Figures 3a and b respectively recorded the depletion rate of CZ48 and the formation rate of CPT in HLM and MLM. As shown in Figure 3A, the depletion rate of CZ48 in MLM was faster than that in HLM in the presence of UDPGA with a total CZ48 recovery of 75.0% in MLM and 92.3% in HLM. The CPT formation rate in MLM was 2.2–3.3 fold higher than that in HLM (Figure 3b).

Compared to Figure 1a, Figure 2a clearly shows that the formation rate of the metabolite CPT in HLM and MLM was respectively 58.36 and 4.3 fold higher in the presence of NADPH than in the absence of NADPH, suggesting that CZ48 metabolism may be proceeded by a CYP enzyme(s)-mediated biotransformation pathway. It was also found that the formation rate of CPT in HLM was 5.21 times higher than that in MLM in the presence of NADPH, which was the opposite of what happened in the absence of NADPH. Interestingly, in HLM, an Eadie-Hofstee curve for CPT formation was obtained when the rate of metabolism was plotted *versus* the concentration of CZ48 in the range from 0.15 to 5 μM as shown in Figure 2b, clearly indicating auto-activation kinetics with the calculated *K*_m_ and *V*_max_ values of 0.42 ± 0.16 μM and 2.2 ± 0.056 nmol/min/mg, respectively. The atypical kinetic profiles displayed herein may not only be substrate-dependent but also enzyme source-dependent. CPT formation exhibited biphasic kinetics, which was characterized by an initial Michaelis-Menten-like increase in velocity as the substrate concentration increased. This resulted in an inaccurate apparent *V*_max_ but an apparent *K*_m_. On the other hand, 99% of CZ48 was depleted within 2 h from human microsomal incubation in the presence of NADPH with a degradation half-life of 19 min, which was estimated by one-phase exponential decay non-linear regression analysis of the degradation time course data. Apparently, several P450 isoforms may be involved in microsomal metabolism as indicated by the observed kinetic behavior. Thus, there is clearly a species difference in the metabolism of CZ48 in liver microsomes between mice and humans.

**Figure 4:**
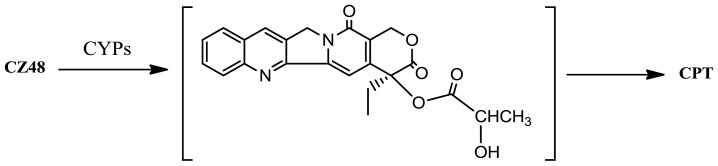
CYP-mediated metabolism of CZ48 to CPT.

In the presence of NADPH, the formation rate of the metabolite CPT in human liver microsomes was significantly higher (*p* < 0.05) than that in mouse liver microsomes with mean *K*_m_s of 1.9 and 0.5 nM and *V*_max_s of 9.3 and 2.2 pmol/min/mg, respectively. However, the apparent intrinsic clearance ratios (*V*_max_/*K*_m_) for CPT metabolism in human and mouse liver microsomes were not significantly different from each other (4.9 *versus* 4.4) (*p* > 0.05). The depletion of CZ48 in human microsomes was multiple times faster with 4.55% of the CZ48 remaining intact compared to the mouse in which 19.11% of the drug unchanged after 60 min. In the absence of NADPH, the depletion of CZ48 in mouse liver microsomes was faster than that in human liver microsomes. These results suggest that there is a remarkable species difference in CZ48 metabolism in liver microsomes between human and the mouse, and also that the greater depletion rate of CZ48 in human liver microsomes is probably due to the involvement of human cytochrome P450s (CYPs); that is, the CZ48 metabolism in the human liver is probably a CYP-mediated process. CZ48 is designed to protect the lactone moiety of the molecule while circulating in the body. When entering into tumors, the drug reacts with tissue esterase(s) to release the active CPT. CZ48 was previously proven to be stable in human plasma [9]. The unexpected low concentration of CZ48 and the high concentration of the metabolite CPT detected in the blood of patients relative to the mouse are because the majority of the CZ48 absorbed by humans is biotransformed into CPT in their liver due to CYP-mediated metabolism. This CYP-mediated reaction may be depicted in the following way (Figure 4):

The CYP-mediated hydroxylation occurs at the α-position of the side chain of CZ48. This α-hydroxylation leads to a less stable intermediate product (compared to the parental CZ48), which could be easily cleaved at the original ester bond (*i.e.*, the bond between 20-*O* and the acyl group) to yield the metabolite CPT. Unlike CZ48, CPT is not stable in human blood mainly because of the affinity of the carboxylate form of CPT to human serum albumin (HSA) [10]. The lactone form of CPT has great anticancer activity. When the lactone of the CPT molecule opens to become a carboxylate, the agent loses 90% of its anticancer activity [11]. Protecting the lactone moiety of the molecule is thus critical to the success of the treatment of cancers in humans.

UGT isoenzymes, expressed in various tissues including liver and intestine, catalyze phase II metabolic biotransformation. It has been widely reported that UGT activity also demonstrates species differences between mice and humans in some common metabolic sites such as liver, lung, and especially intestine. Figures 3A and 3B clearly show a metabolic difference in liver microsomes between mouse and man, but the difference was not as dramatic as the CYP case. The depletion of CZ48 in HLM was somewhat slower than in MLM, implying that some specific UGT isoforms in MLM may be responsible for catalyzing CZ48 biotransformation to CPT a bit faster. Overall, the UGT effect on the metabolism of CZ48 in HLM and MLM was minimal.

## 3. Experimental Section

### 3.1. Chemicals

HPLC-grade acetic acid, dimethyl sulfoxide (DMSO), acetonitrile, dichloromethane and diethyl ether were obtained from Sigma-Aldrich (St. Louis, MO, USA). Chromatographic-grade water was produced by a Millipore Milli-Q System (Billerica, MA, USA). CZ48 and CZ44 (internal standard for HPLC quantification) were synthesized in-house by using reported procedures [12]. Camptothecin (with a purity of 99%) was purchased from China and used without further purification.

### 3.2. CYP Activity

The activity of CYP enzymes was evaluated by a method reported by Fujino and his co-workers [13,14]. Briefly, CZ48 was incubated with pooled human liver microsomes purchased from Sigma for up to 2 h at 37 °C at a concentration of 1 mg/ml microsomal protein. All incubations were finally performed in a 1 mL reaction solution containing 1.3 mM NADPH, 3.3 mM glucose-6-phosphate, 0.4 U/mL Glucose-6-phosphate dehydrogenase, and 3.3 mM MgCl_2_. The reaction started by adding the microsomal protein solution following 3 to 5 min of prewarming. The reaction stopped by adding acetonitrile at the designated time. The mixture was extracted and the combined extracts were analyzed by HPLC following a reported procedure [15].

### 3.3. UGT Activity

The activity of UGT was evaluated in the following way: all incubated microsomes were treated with alamethicin (50 ng alamethicin/mg microsomal protein), 10 mM magnesium chloride, 25 mM saccharolactone and CZ48 in 50 mM KPI buffer (pH 7.4) for 3 min at 37 °C. The reactions initiated by the addition of 25 mM UDPGA were allowed to proceed for 120 min at 37 °C and were then stopped by the addition of 50 μL 94% acetonitrile/6% glacial acetic acid containing 100 μg/mL cortisone as an internal standard. The mixture was extracted and the combined extracts were then analyzed by HPLC following our established method [15].

### 3.4. Kinetic Data

Kinetic data (*K*_m_ and *V*_max_) were obtained using the Michaelis-Menten equation: *V* = *V*_max_ × *S*/(*K*_max_ + *S*), where *V* is reaction rate, *S* is substrate concentration, and *V*_max_ represents the maximum rate achieved by the system, at maximum (saturating) substrate concentrations. The Michaelis constant *K*_m_ is the substrate concentration at which the reaction rate is half of *V*_max_. The Curve was drawn fitting with simple weighting, and the least-squares minimization procedure (regression analysis) was operated using Excel Solver.

## 4. Conclusion

The new anticancer agent CZ48 metabolizes in human liver microsomes much faster than in mouse liver microsomes in the presence of NADPH and slower in the absence of NADPH, indicating different metabolic pathways in humans *versus* mice. In human liver, the CYP isoenzymes play a major role in catalyzing the formation of CPT from CZ48. In order to maintain an effective concentration of CZ48 circulating in the human body, efforts are required for identifying the individual CYP isoenzymes responsible for the α-hydroxylation at the side chain of CZ48, and, subsequently, a corresponding method for inhibiting this CYP-mediated metabolic process is needed. The effects of UGT isoenzymes on the metabolism of CZ48 in human and mouse liver microsomes exist, but are not significant. These results are useful for further development of CZ48 for clinical use, especially in designing the CZ48 dosing strategies for current and future human clinical trials. The study of the biotransformation of CZ48 in other organs and the identification of specific CYP (s) responsible for the discussed liver microsomal metabolism are to be performed and will be reported subsequently.
